# Prevalence of lipodystrophy and metabolic syndrome among HIV positive individuals on Highly Active Anti-Retroviral treatment in Jimma, South West Ethiopia

**Published:** 2012-10-30

**Authors:** Tsegay Berhane, Alemishet Yami, Fessahaye Alemseged, Tilahun Yemane, Leja Hamza, Mehedi Kassim, Kebede Deribe

**Affiliations:** 1 Jimma University, faculty of medicine, Internal medicine department, Jimma, Ethiopia; 2 Jimma University, Facility of Public health Department of Epidemiology and Biostatistics, Jimma, Ethiopia; 3 Brighton and Sussex Medical School, Falmer, Brighton, United Kingdom

**Keywords:** HIV-lipodystrophy, metabolic syndrome, anti-retroviral

## Abstract

**Introduction:**

Use of highly active antiretroviral therapy has led to significant reductions in morbidity and mortality rates. However, these agents had also given rise to the metabolic and morphologic abnormalities which are modifiable risk factors for cardiovascular diseases. Evidences elsewhere indicate growing in prevalence of these problems but studies are lacking in Ethiopia. This study was conducted to determine the prevalence of HIV-associated lipodystrophy and metabolic syndrome in patients taking highly active antiretroviral therapy.

**Methods:**

A cross-sectional study was conducted in 2010 on a sample of 313 patients taking highly active antiretroviral therapy in Jimma University specialized hospital. Structured questionnaire was used to assess patients’ sociodemographic characteristics and clinical manifestations of metabolic abnormalities. Checklists were used for reviewing charts about clinical manifestations of metabolic abnormalities and immunologic profile of patients. Data was cleaned, entered in and analyzed using SPSS for windows version 16.0.

**Results:**

Metabolic syndrome was detected in 21.1% and HIV-lipodystrophy was detected 12.1% of patients. The factors found to be independently associated with metabolic syndrome were taking the antiretroviral therapy for more than 12 months (AOR=4.2; 95% CI=1.24–14.23) and female sex (AOR=2.30; 95% CI=1.0–5.27) and the factor found to be independently associated with HIV-lipodystrophy was taking the antiretroviral therapy (AOR=3.59; 95% CI=1.03–12.54) for more than 12 months.

**Conclusion:**

Metabolic abnormalities were relatively common in the study population. The problems were higher among those who took anti-retroviral treatment for longer duration. Therefore, regular screening for and taking action against the metabolic abnormalities is mandatory.

## Introduction

The advent of HARRT is a breakthrough in improving longevity and quality of life of PLHIV. However the treatment is not without risk or side effects. Dyslipidemia is one of the complications of highly active antiretroviral therapy (HAART), and it has a well established linked to an increased risk for cardiovascular morbidity in HIV infected individuals [1]. Patients with HIV infection receiving HAART also, develop a syndrome referred to as HIV-associated lipodystrophy, consisting of elevations in plasma triglycerides, total cholesterol, and hyperglycemia. The treatment of patients with HIV infection requires not only a comprehensive knowledge of ARV therapy, but also the ability to deal with the problems of a chronic, potentially life-threatening illness like cardiovascular diseases [2–5].

Among HIV-infected patients taking HAART there are reports of adverse effect treatment from different countries [6, 7] particularly like HIV-associated lipodystrophy and metabolic syndrome. Few studies have been able to directly assess potential associations between metabolic syndrome and the incidence of coronary heart disease (CHD) in HIV-infected patients [8]. In developing countries like Ethiopia no or very few research is done to assess ARV treatment with HIV-associated lipodystrophy and metabolic syndrome. Therefore this study was conducted to estimate the prevalence of lipodystrophy and metabolic syndrome among HIV-infected patients taking HAART. The findings generated from this study may make contributions to both knowledge and understandings of the magnitude of HIV-associated lipodystrophy and metabolic syndrome in those taking HAART in the area.

## Methods

### Study area and period

Jimma University specialized Hospital (JUSH) is the only hospital in Jimma zone serving the majority of people living in Jimma town and its surrounding. JUSH provides both inpatient and outpatient services and as one of the inpatient services. JUSH ART service was started 2003 with fee and on September 2005 free ART service was started. The total number who ever enrolled in ART service were 6500 (pre ART and ART) and who ever started HAART were 3003 of these who were active during the study period 1876 (all adults and children) of these 1684 were adults. The study was conducted from September 15, 2010 to December 10, 2010.

### Study population and design

This is a cross-sectional study facility based study which employed quantitative methods. The source population for the study was all adult HIV patients who were on HAART during the study period in JUSH ART clinic and age greater than 18yrs (1684). The study population all clients sampled from the source population who fulfilled eligibility criteria were included in the study. The inclusion criteria were age ≥18 years and those who were adhering to and taking HAART for at least 6 weeks before the study period. Six weeks was taken as a criterion because the earliest time to develop HIV associated lipodystrophy or metabolic syndrome was seen since 6 weeks of initiation of ART [2]. The exclusion criteria were withdrawal from combination ART since the objective of the study was to assess the prevalence HIV lipodystrophy and metabolic syndrome in those who were on ART taking with good adherent during the study period, patients with hypothyroidism, Diabetes mellitus, chronic Renal Failure and those using corticosteroids.

### Sample size and sampling technique

The samples size was estimated a single population proportion formula with finite population correction was used. The following parameters were considered 50% prevalence, 95% confidence interval, 5% margin of error. The size of the source population obtained from the ART clinic was 1694. With the above assumptions, the calculated sample size was 313.

### Sampling technique

As duration of ART intake was known to affect the prevalence of HIV lipodystrophy, clinical lipodystrophy and metabolic syndrome a stratified sampling was used. The source population was stratified into two as those who took HAART for less than 6 months and those who took more than 6 months. The reasons for taking 6 months for stratification are the fact that CD4 count which affects the dependent variables was measured every 6 months and also other literatures this cut-off was used and hence valid comparisons can be made. Then sample was allocated to the strata proportional to their size. So the sample size was made 278 from clients on ART follow up for more than 6 month and 35 clients from those on ART follow up for less than 6 month. All patients fulfilling the eligibility criteria who come to the ART clinic was sequentially included in the study till the sample size allotted to each stratum was reached. The sampling technique employed was sequential method.

### Measurement

The dependent variable are presence of clinical lipodystrophy (fat accumulation, fat wasting), presence of metabolic syndrome, presence of dyslipidemia. The independent variables are age, sex, CD4 count, WHO staging, type of HAART regimen, and duration on HAART.

For this study hypertension was defined by - systolic BP of ≥140 mmhg and/or diastolic pressure of ≥ 90 mmgh or being on anti- hypertensive medication [9]. Major Risk Factors for coronary heart disease (CHD) in this study include cigarette smoking, hypertension, low plasma HDL-c level, and family history of premature (CHD), age and diabetes mellitus. Cigarette smoking designation “smoker” means any cigarette smoking in the past month. Low plasma HDL-c was defined as HDL-c level <40 mg/dl. Family history of CHD refers to male first degree relative <65 Years with CHD.

Adult Treatment Panel III criteria [9] were used in this study, and MetSynd was diagnosed when 3 or more of the following were present: abdominal obesity (waist circumference men, >102 cm; women, >88 cm), hypertriglyceridemia of 150 mg/dL or higher, low high-density lipoprotein (HDL) cholesterol (men, <40 mg/dL; women, <50 mg/dL), blood pressure (BP) of 130/85 mm Hg or higher or current use of antihypertensive medication, and fasting glucose of 100 mg/dL or higher, or previously diagnosed diabetes mellitus.

Dyslipidemia defined as presence of one or more of the following; Hypercholesterolemia, serum TC levels ≥240 mg/dl, Low HDL-c, serum HDL-c levels <40 mg/dl, High TC: HDL-c ratio, serum TC: HDL-c ratio ≥4.5, High LDL-c, serum LDL-c levels ≥160mg/dl and Hypertriglyceridemia serum fasting TG levels ≥200 mg/dl [10].

Clinical Lipodystrophy defined as peripheral lipoatrophy with or without central obesity. Central obesity is excess fat deposition in the abdominal viscera or supraclavicular fossae or in the posterior neck. Fat wasting consists of subcutaneous fat atrophy in the legs, buttocks, arms, face [11]. Body Mass Idex is weight in kilograms divided by height in meters squared. Values 18.5–24.9 are normal, <18.5 underweight, 25–29.9 are overweight, ≥30 are obese [12]. Plasma glucose level was measured according to the following classification: normal- fasting plasma glucose [13]. HIV Lipodystrophy was measured according to measurements from previous studies [14, 15].

Clinical Lipodystrophy defined as peripheral lipoatrophy with or without central obesity. Central obesity is excess fat deposition in the abdominal viscera or supraclavicular fossae or in the posterior neck. Fat wasting consists of subcutaneous fat atrophy in the legs, buttocks, arms, face [11]. Body Mass Idex is weight in kilograms divided by height in meters squared. Values 18.5–24.9 are normal, <18.5 underweight, 25–29.9 are overweight, ≥30 are obese [12]. Plasma glucose level was measured according to the following classification: normal- fasting plasma glucose [13]. HIV Lipodystrophy was measured according to measurements from previous studies [14, 15].

### Data collection instruments

Data was collected by using structured questionnaire and checklists. The questionnaire was prepared in English and translated to local languages (Afan Oromo and Amharic) and then back translated to check consistency. The questionnaire was developed by the investigators based on the study objectives and was pre-tested on 5% of the sample. The questionnaire contains questions about socio demographic and clinical variables. The checklists were used for chart review and observation. Chart review checklist was used to collect data concerning WHO staging, and immunologic variable including CD4 count. Observational checklist was used for recording laboratory measure about lipid profile and fasting blood sugar.

### Data collection process

Interview and physical examination was conducted by internal medicine residents using interview for demographic and clinical characteristics like obesity and wasting and the physician performed physical examination to confirm patient report of wasting and obesity because this was the preferred method for describing wasting and obesity as there was no standard criteria used to assess HIV-lipodystrophy [11]. Review of patients charts obtained from the ART clinic was carried out by HIV/AIDS nurse specialist (HANS). The review was done to record WHO staging and CD4 count.

Blood sample was collected from the participants on the next morning after fasting for 12 hours to determine fasting serum glucose and lipid profile. Five milliliter of blood was drawn from each patient from cubital vein for each subject. Laboratory examination was done by a professional who has masters in laboratory methods and has relevant experience.

### Data analysis and interpretation

Data was cleaned, coded and inserted in computer using SPSS 16.0 for analysis. Descriptive statistics using frequency distribution was performed for socio-demographic, epidemiological, clinical, and laboratory values. The prevalence of HIV-lipodystrophy and metabolic syndrome were determined based on the definition used in the study. Low-density lipoprotein (LDL) cholesterol was calculated by the Friedewald method, except in patients with TG levels higher than 400 mg/dL (LDL=CHOL - ({TG/5} + HDL). The association between the independent and dependent variables were assessed using chi-square test and logistic regression model. Multivariate analysis using logistic regression was performed to control effect of confounding variables.

### Data quality assurance

The following measures were undertaken to assure quality of data: Before data collection the internal medicine residents were trained by the principal investigator for 1 day on the objectives of the study, interviewing and physical examination using practical scenario. HANS nurse was trained for 1 day on chart review contents. For laboratory examination, a qualified professional who has masters in laboratory and relevant experience were recruited. The laboratory personnel were also given orientation by one of the investigators who have the qualification and experience on related laboratory measurements. Serum glucose and serum lipid was analyzed using clinical chemistry analyzer Humastar 80 made in GERMANY in 2009 and approved by Ethiopia Health and Nutrition Research Institute (EHNRI). The data collection instruments were pre-tested in JUSH ART clinic on 5% of the sample and necessary modifications were made based on results of the pre-test. The pre-tested cases were not included in the final report. The questionnaire was translated to local languages and back translated by different individuals to assure consistency. The laboratory reagents were assured by the University Laboratory School. Laboratory machine used for the study was approved by the Science and Technology currently using for the teaching research purpose by the University.

### Ethical considerations

The study was undertaken after ethical approval from Jimma University Ethics Review Committee. Letter was given to the Jimma University Specialized Hospital and after permission secured from the hospital data collection was started. Written, informed consent was obtained for all participating subjects in the local language prior to interview during the whole study period. Data obtained during the study was treated as confidential and the records were kept in a locked cabinet. The participant had the given full right to refuse participating in the study. Patients with newly diagnosed with metabolic syndrome and HIV-lypodystrophy was counseled on the ongoing risks to cardiovascular complications and was referred to cardiac clinic for treatment and follow-up.

## Results

### Patient characteristics

In this study the response rate was 100%. Of the total of 313 included in the study the majority (65.2%) are females. Concerning the age category most are in age category between 30–39years (40.9%) followed by the age groups 18–29years (33.9%) and above 40years (25.2%). With regarded to ethnicity majority of the study population are Oromo (43.1%). The predominant Religion in the study was orthodox that comprised 51.4%, followed by muslin and protestant, (30%), (16.9%) respectively. With regard to marital status the majority were married (54.3%). Concerning the literacy status of the study population the majority were 1–6 graders (31.3%) followed by 10–12 graders (25.9%) and 7–9 graders (16.6%). Regarding the occupation status of the study population majority were daily laborer (24.6%) and Government employee (23.3%). Concerning the monthly income in Ethiopian Birr; majority were getting less than 250 Birr (43%). Hypertension was found in (16.0%) (Table 1).

**Table 1:** Baseline characteristic of patients on HAART, Jimma University Specialized Hospital 2010; Jimma

Variable		Number	Percent
**Sex**	Female	204	65.2
	Male	109	34.8
**Age in years**	18-29	106	33.9
	30-39	128	40.9
	>40	79	25.2
**Ethnicity**	Oromo	135	43.1
	Amhara	93	29.7
	Kefa	35	11.2
	Gurage	18	5.8
	Others§	32	10.2
**Religion**	Orthodox	161	51.4
	Muslim	94	30
	Protestant	53	16.9
	Others£	5	1.6
**Marital status**	Single	56	17.9
	Married	170	54.3
	Divorced	31	9.9
	Widowed	38	12.1
	No response	18	5.8
**Education**	Illiterate	48	15.3
	1-6 grade	98	31.3
	7-9 grade	52	16.6
	10-12 grade	81	25.9
	12+ grade	34	10.9
**Occupation**	Unemployed	60	19.2
	Employed	253	80.8
**Monthly income**	<250	116	43
	251-500	86	31.9
	501-1000	18	6.7
	>1000	50	18.5
**Hypertension**	≥ 140 and /or ≥ 90	50	16

§ Tigre, Wolaita and Yem

£ Catholic and traditional

### Prevalence and factors associated with HIV lipodystrophy

The factors that were used to assess presence of HIV lipodystophy were listed in Table 2. Based on the criteria used in the study HIV-lipodystrophy was found in 38(12.1%) patients (30 females, 8 males).

**Table 2:** Prevalence of criteria used for HIV lipodystrophy of HIV patients on HAART, Jimma University Specialized Hospital 2010; Jimma

Variable	Number	Percent
Fat wasting	52	16.6
Central obesity	43	13.7
Total cholesterol ≥ 214mg/dl	43	13.7
Triglyciredemia ≥ 174mg/dl	83	26.5
Fasting glucose ≥ 100mg/dl	78	24.9

As indicated in Table 3 the only factors independently associated with HIV LD was only taking HAART for more than a year (AOR=3.59; 95% CI=1.03–12.54) (Table 3).

**Table 3:** HIV Lipodystrophy and associated factors of HIV patient on HAART Jimma University Specialized Hospital 2010; Jimma

Variable	Presence of HIV- lipodystrophy		COR (95% CI)	AOR (95% CI)
	Yes	No		
**Sex**				
Male	8 (7.3%)	101 (92.7%)	2.18 (0.96-4.93)	2.32 (0.98-5.49)
Female	30 (14.7%)	174 (85.3%)	1.0	1.0
**Age in years**				
18-29	14 (13.2%)	92 (86.8%)	1.0	1.0
30-39	15 (11.7%)	113 (88.3%)	1.18 (0.48-2.9)	0.94 (0.42-2.09)
≥40	9 (11.4%)	70 (88.6%)	1.03 (0.42-2.28)	1.13 (0.44-2.94)
**Duration HAART**				
<12month	3 (3.8%)	75 (96.2%)	1.0	1.0
>12month	35 (14.9%)	200 (85.1%)	4.37 (1.30-14.70)	3.59 (1.03-12.54)
**WHO staging**				
<3WHO stage	13 (9.6%)	123 (90.4%)	1.56 (0.8-3.17)	1.40 (0.67-2.91)
≥3WHO stage	25 (14.1%)	152 (85.9)	1.0	1.0
**Initial CD4**				
<200	28 (13.0%)	188 (870%)	0.65 (0.28-1.49)	
≥200	8 (8.90%)	82 (91.1%)	1.0	-
**Regimen**				
D4T Exposed	31 (14.6%)	182 (85.4%)	0.44 (0.19-1.04)	0.61 (0.25-1.49)
Non D4T Exposed	7 (7%)	93 (93%)	1.0	1.0

### Prevalence and factors associated with metabolic syndrome

Hypertriglyceridemia were detected in 39%, hypertension in 35.1%, impaired fasting plasma glucose in 24.9%, low HDL cholesterol in male 40(36.7%), low HDL cholesterol in female 109(53.4%) and abdominal obesity was observed only in females 19(9.3%) but there was no male seen with abdominal obesity in the study (Table 4). As can be seen from Table 5 based on the criteria used in the study metabolic syndrome was found in 66(21.1%). The factors found to have independent statistically significant association with metabolic syndrome were taking HAART for more than a year (AOR=4.2; 95% CI=1.24–14.23) and female sex (AOR=2.30; 95% CI=1.0–5.27.)

**Table 4:** Criteria used for metabolic syndrome of HIV patients on HAART, Jimma University Specialized Hospital 2010; Jimma

Variable	Number	Percent
Fasting triglycerides ≥150mg/dl	122	39
Impaired fasting glucose ≥ 100mg/dl	78	24.9
Hypertension ≥130 and/or ≥ 85 or history of hypertension	110	35.1
**HDL**		
Female <50mg/dl	109	53.4
Male <40mg/dl	40	36.7
**Abdominal circumference**		
Female >88cm	19	9.3
Male >102cm	–	–

**Table 5:** Metabolic Syndrome of HIV patient on HAART, Jimma University Specialized Hospital 2010; Jimma

Variables	Presence of Metabolic Syndrome		COR (95% CI)	AOR (95% CI)
	YES	NO		
**Sex**				
Male	19 (17.4%)	90 (82.6%)	1.41 (0.78-2.56)	2.30 (1.0-5.27)
Female	47 (23.0%)	157 (77.0%)	1.0	1.0
**Age in years**				
18-29	16 (15.1%)	90 (84.9%)	1.0	
30-39	25 (19.5%)	103 (80.5%)	0.38 (0.88-0.78)	-
≥40	25 (31.6%)	54 (68.4%)	0.52 (0.27-0.99)	
**Duration ART (months)**				
<12	10 (12.8%)	68 (87.2%)	1.0	1.0
≥12	56 (23.8%)	179 (76.2%)	2.12 (1.02-4.40)	4.20 (1.24-14.23)
**WHO staging**				
Stage I & II	32 (23.5%)	104 (76.5%)	0.77 (0.44-1.33)	1.40 (0.67-2.92)
Stage III & IV	34 (19.2%)	143 (80.8%)	1.0	1.0
**Initial CD4**				
<200	48 (22.2%)	168 (77.8%)	0.70 (0.36-1.32)	
≥200	15 (16.7%)	75 (83.3%)	1.0	-
**Regimen**				
D4T EXPOSED	48 (22.5%)	165 (77.5%)	0.75 (0.41-1.37)	
NON D4T EXPOSED	18 (18.0%)	82 (82.0%)	1.0	–

### Dyslipidemia

Prevalence of dyslipidemia from 313 of the study population of any type with one type or more was seen in 48.2% (Table 6). Concerning the causes of dyslipidemia the most common types of dyslipidemia was low HDL-c level (32.6%), followed by high ratio of total cholesterol to high density lipoprotein cholesterol (≥4.5) in (25.6%), elevated triglycerides cholesterol level in (18.2%), high LDL-c level in (6.9%), high total cholesterol level in (6.7%) (Table 6).

**Table 6:** Lipid profile of HIV patients on HAART, Jimma University Specialized Hospital 2010; Jimma

Variables	Values	Number	Percent
**Total cholesterol(mg/dl)**	<240	292	93.3
	≥240	21	6.7
**Triglycerides(mg/dl)**	<200	256	81.8
	≥200	57	18.2
**LDL(mg/dl)** ^**¶**^	<160	283	93.1
	≥160	21	6.9
**HDL(mg/dl)**	<40	102	32.6
	≥40	211	67.4
Ratio TC:HDL*	<4.5	233	74.4
	≥4.5	80	25.6
**Total with dyslipidemia**	–	151	48.2

* Ratio of total cholesterol to high density lipoprotein LDL level was not measured for nine patients due to their triglycerides was >400mg/dl

## Discussion

This study identified many important findings and factors associated with metabolic disorders among PLHIV taking HAART. The prevalence of HIV-lipodystrophy was found to be 12.1% and duration of HAART for more than a year was found to be significantly associated factor. The prevalence of Metabolic Syndrome was found to be 21.1% and duration of HAART for greater than a year and female sex were associated with Metabolic Syndrome. The prevalence of dyslipidemia in our study was found to be 48.2%. The implications of the results are discussed based on previous findings.

In our study fat wasting was seen in 16.6% and central obesity in 13.7% of the study population. This is similar with reports for fat redistribution which ranging from 14% to 32% in HIV-positive population on HAART [16]. A study done in Spain in 156 patients on HAART clinical lipodystrophy was present in 63.4% of cases, of which 26.3% with central obesity [17]. This was higher than the prevalence seen in our study this could be due to the inclusion criteria they used: they included those patients on HAART for six months however in our study we included all patients taking HAART since six weeks.

Prevalence of HIV-lipodystrophy in the study is 12.1%. In our study the only significantly and independently association with HIV lipodystrophy was taking HAART for more than a year, previous studies have identified other factors [18]. Prevalence of hypertension in our study was found 16.0%. A study done in patient on HAART in Kenya documented a prevalence of 13.4% [19].

In our study the prevalence of MS was 21.1%, this finding is higher than the prevalence of MS reported in Spain 15.8% [17], comparable with 20.8% Italian study [20] and lower than another Italian study [21] which reported 25.4%. The latter Italian study was conducted in those patients on HAART for more than 6 months; however in our study we included all patients on HAART. In our study factors found to have independently associated with MS were taking HAART for more than a year and female sex. A recently published large epidemiological study has found several factors related to an increased risk for MS in HIV-infected patients [17]. The effect of treatment duration is also documented elsewhere [22].

Prevalence of dyslipidemia in our study is 48.2% the most common types being low HDL-c level in 32.6%. In our study total cholesterol, triglycerides, HDL level were lower than the study done in Tehran [23]. The reason for the lower level in our study could be no patient was taking PI during our study.

The level of low density lipoprotein for nine people was not determined because the value of triglyceride level was more than 400mg/dl, because the formula we used in the study for calculating LDL based on Total cholesterol, triglyceride and HDL level is validated for those serum triglyceride level of less than 400 mg/dl and recommends exclusion of patients having higher level. In light of these limitations we conclude that fat redistribution, HIV associated lipodystrophy, metabolic syndrome, dyslipidemia are common in those HIV-infected patients receiving HAART in JUSH. It is proven that CVD risk will be high especially in patients with metabolic syndrome this may put the patient at high risk of CVS.

## Conclusion

Metabolic syndrome was detected in 21.1% and HIV-lipodystrophy was detected 12.1% of patients. Metabolic abnormalities were relatively common in the study population. The problems were higher among those who took anti-retroviral treatment for longer duration. This puts them at high risk of cardiovascular morbidity and mortality. Therefore, patients with HIV infection on HAART should be screened for lipid disorders, given their incidence, potential for morbidity, and possible long-term cardiovascular risk. Initial screening for fasting blood glucose and lipid profile blood before instituting therapy and should include measurement of fasting TC, HDL-C, and TG levels. Encourage regular exercise, provision of lipid lowering agents for those with metabolic syndrome and dyslipidemia. Further wide scale study is recommended to evaluate the long term impact of HAART on cardiovascular risks and associated outcomes.
